# Community Education for a Dignified Last Phase of Life for Migrants: A Community Engagement, Mixed Methods Study among Moroccan, Surinamese and Turkish Migrants

**DOI:** 10.3390/ijerph17217797

**Published:** 2020-10-24

**Authors:** Xanthe de Voogd, Dick L. Willems, Bregje Onwuteaka-Philipsen, Marieke Torensma, Jeanine L. Suurmond

**Affiliations:** 1 Amsterdam UMC, Department of Public & Occupational Health, Amsterdam Public Health Research Institute, University of Amsterdam, Meibergdreef 9, Postbus 22660, 1100 DD Amsterdam, The Netherlands; m.torensma@amsterdamumc.nl (M.T.); j.suurmond@amsterdamumc.nl (J.L.S.); 2 Amsterdam UMC, Department of Ethics, Law and Humanities, Amsterdam UMC Expertise Center for Palliative Care and Amsterdam Public Health Research Institute, Meibergdreef 15, 1105 AZ Amsterdam, The Netherlands; d.l.willems@amsterdamumc.nl; 3 Amsterdam UMC, Department of Public & Occupational Health, Amsterdam UMC Expertise Center for Palliative Care and Amsterdam Public Health Research Institute, Van der Boechorststraat 7, 1081 BT Amsterdam, The Netherlands; b.philipsen@amsterdamumc.nl

**Keywords:** migrants, minority groups, access to health care, community engagement, community health education, palliative care, end-of-life, dignity

## Abstract

Community engagement and -education are proposed to foster equity in access to care and to ensure dignity of migrant patients in the last phase of life, but evidence is lacking. We evaluated nine community educational interactive meetings about palliative care (136 participants totally)- co-created with educators from our target groups of Moroccan, Surinamese and Turkish migrants—with a mixed methods approach, including 114 questionnaires, nine observations, nine interviews with educators, and 18 pre- and post- group- and individual interviews with participants. Descriptive and thematic analysis was used. 88% of the participants experienced the meetings as good or excellent. Educators bridged an initial resistance toward talking about this sensitive topic with vivid real-life situations. The added value of the educational meetings were: (1) increased knowledge and awareness about palliative care and its services (2) increased comprehensiveness of participant’s wishes and needs regarding dignity in the last phase; (3) sharing experiences for relief and becoming aware of real-life situations. Community engagement and -education about palliative care for migrants effectively increases knowledge about palliative care and is a first step towards improved access to palliative care services, capacity building and a dignified last phase of life among migrants.

## 1. Introduction

Equality and equity in access to care are generally agreed to be needed to preserve human dignity of all persons [1]. Across Europe, including in The Netherlands, studies show that access to palliative care services is unequal and hampered for migrant patients and their families [2,3]; they make less use of palliative care services or services in which palliative care is provided, such as hospice care, nursing home care and home care [4,5,6]. A dignified last phase of life might therefore be at stake for these patients. Studies show a combination of factors relate to this limited use of palliative care services, namely a lack of capacity of home care services and hospices to address needs of migrants patients and their families, and limited knowledge, negative images about home care and struggles to find homecare due to language barriers among migrants [4,7,8,9].

Besides these practical and social factors that limit the access to palliative care services, tensions between preferences and expectations among migrant patients and their families and standard ways of work in palliative care are also found. In many health care facilities across Europe, especially in Western-European countries such as the Netherlands, it is customary to openly discuss the last phase of life [10], to involve the patient as the main decision maker, and to have a certain control over the end of life [11]. This conflicts with preferences for not wanting to talk about the last phase of life; nondisclosure of diagnosis or prognosis and an indirect communication style [12,13,14]; high involvement of family in decision making; and the belief that God or Allah decides about life and death, and a preference for maximum curative treatment [11,15,16,17,18,19]. These preferences are found in several studies about palliative care for migrant patients but may also apply to non-migrant patients and their families.

From our study among migrant patients in The Netherlands [9], we know above described conflicts can influence the sense of dignity of migrants, because these preferences are related to what is regarded as dignified by migrant patients and their families. Religion and surrender to God or Allah can be important for dignity among migrant patients, as well as (completely) being cared for or assisted by family members. When this cannot be accomplished or when abovementioned preferences cannot be met, this creates barriers for optimal use and experienced satisfaction of palliative care services and may lead to situations in which the experience of dignity is at stake. There is a need to know among migrant families about how to shape care in the last phase of life among family members in the context of existing palliative care facilities, and still maintain the patient’s dignity [9].

To improve access to palliative care and optimal use of services, community education about palliative care and its services is proposed as an important strategy [20,21]. However, studies on community education among migrant patients are scarce and the ones carried out mainly focused on giving information about and support with advanced care planning for patients and/or their families [22,23,24]. These interventions do not address aspects important for the sense dignity of migrant patients, such as care by family, not openly discussing the last phase of life or family decision making. Given the abovementioned literature, addressing these themes in community education is necessary to empower migrant patients to know how to act upon personal needs and wishes for a dignified last phase of life. Evidence-based community education is scarce, and therefore we developed and evaluated community education (see Box 1) with the aim to provide participants with tools and information to discuss important subjects for their dignity and to know how they can preserve their dignity in the context of palliative care services.

Box 1 **Development of the community education.**

We took a community engagement approach, to develop, recruit for and execute the community education. Community engagement serves to build capacity and addresses matters important to the community. It can foster development of a community support structure encompassing community members who gain and share knowledge among the community [25,26,27]. For us this was a useful method, because it met our aim to ascertain dignity in the last phase of life, by addressing the needs of the community. We co-developed the educational sessions with ethnic-matched community educators from migrant network organisations who were engaged from the start of our project. We worked together as equals and synthesized knowledge based on previous study findings among patients with similar ethnic backgrounds as well as the expertise of the educators about (other) relevant themes within their communities and their knowledge and experience with community education about health related subjects. During this process, we learned from the knowledge and experience from the educators, and the educators learned from the knowledge from previous study findings that we presented.

Co-development took place in two sessions. In the first session we brainstormed about important themes and elements for the educational sessions and XV presented the main themes from the literature and interviews with patients and family from the same ethnic groups from an earlier stage in our project [9]. This served the development of community education suiting the target groups, by XV and JS. In the second session XV presented the education and, together with the educators and JS, discussed questions and practical matters for execution.

Aims and content of the educational meeting

The general aim of the meetings was to increase the ability to know and act upon wishes in the context of palliative care services and to improve the future experience of palliative care and preserve dignity in the last phase of life. To meet this aim, the education encompassed the following components: (1) information about palliative care and its focus on quality of life; (2) discussion about the wish to know or not to know about diagnosis and prognosis; (3) information and discussion about the patient as the main decision maker; (4) how care (by family) could be shaped in the context of palliative care services; (5) reflection about what would be important in the last phase of life.An additional aim was to get participants familiar with talking about the last phase of life, since being able to talk about this is needed to become aware of own wishes and needs and act on them [13,28,29]. The sessions were therefore aimfully interactive and we used video-material of re-enacted real-life situations developed by a partner organisation [30], yes/no-voting on propositions about the last phase of life, and personal questions to discuss in pairs with other participants. See “Supplementary Materials” for the online videos used in this project.

Recruitment of participants

Most of the participants were recruited by the educators themselves, through phone and personal approaching with a flyer. Generally, they approached and merged groups who they educated before, or they recruited acquaintances from their mosque or church. One educator didn’t know the participants; recruitment took place through someone else from the migrant network organisation she was part of.

We evaluated the community education using the following research questions:

(1) How is the community education executed and experienced by participants and educators?
(2) What is the added value of this community education?

## 2. Materials and Methods

### 2.1. Design

We focused on the three largest migrant groups in the Netherlands which are persons with a Moroccan, Turkish and Surinamese background [31]. The latter consists of two main ethnic groups, the African (Creole) and South-Asian (Hindustani). To gain rich understanding we used a convergent parallel design [32] (a mixed methods design [33]) to evaluate the educational meetings. We collected the different forms of data at the same time, kept the data analysis independent but mixed the results —by comparing them and looking for relationships and contradictions—during the interpretation phase. We gained insight about overall experience and reach of the educational meetings with a quantitative method, the questionnaire, and deeper insight into the experience, manners of execution and the added value of the community education with qualitative methods (see Table 1). We used a combination of qualitative and quantitative methods because of unfamiliarity with questionnaires among many persons from our target groups, confirmed by the educators, and the lack of a standardized evaluation questionnaire. A quantitative pre-test and post-test about knowledge and wishes would therefore not be suitable.

Questionnaires were used to evaluate the experience of the information provided, subjects discussed, the interactive elements and demographics of the participants, mainly with 5-point Likert scales and yes/no questions. Likert scales were presented with smiley faces [34], instead of text, because of illiteracy/numeracy, confirmed by the educators, and unfamiliarity with questionnaires among persons from our target groups. See Appendix A for the questionnaire. The smiley faces represented the choices (1) very poor (2) poor (3) acceptable (4) good (5) very good.

The aim of the meetings was to increase the ability to know and act upon wishes and to improve the experience of palliative care and dignity in the last phase of life. Based on this aim and the components of the educational meetings (see Box 1), we determined that participants would afterwards have an increased knowledge about palliative care, increased ability to describe their own wishes with regard to the subjects we discussed and increased insight into the importance of talking about wishes for the last phase of life. Therefore, as part of determining the added value of the meetings, we performed semi-structured interviews with participants before the meeting, and two-three weeks after, to explore and compare their knowledge, descriptions of wishes, and their perspectives on talking about the last phase of life between before and after the educational meetings. Group interviews were performed before and afterwards to explore knowledge and familiarity with the subject, and experienced added value of the meetings. Observations and semi-structured interviews with the educators served to observe, explore and discuss participant’s responses, educator’s experiences and explanations for their way of execution. Triangulation of methods was performed, for example, by comparing the qualitative outcomes, such as observed participants’ responses, with demographics and satisfaction of the meeting measured with the questionnaire. Table 1 shows all relevant details of the evaluation methods, their purposes and number of participants. See Appendix B and Appendix C for the interview guides and the observation list.

Bilingual ethnically-matched research assistants with a Turkish and Moroccan background from our network [35] did the observations and interviews with the educators together with XV, and performed and led the interviews and groups discussions with the participants with a non-Dutch language preference. Because of the colonial history of the Netherlands with Suriname, most of the Surinamese persons who live in the Netherlands have Dutch as their mother tongue. Therefore we did not need a Surinamese interviewer. XV did the rest of the interviews, observations and group discussions with participants who preferred the Dutch language.

We worked together with educators from our target groups. Three educators had either experience with community education about related subjects, such as cancer and dementia, the three other educators had experience with leading group discussions in an informal or educational setting and had experienced the last phase of life of a loved one. Table 2 shows the characteristics of the educators.

The educational meetings took place in common spaces of elderly communes, community centers, a mosque and a nursing home, and according to language preference of the participants. Nine educational sessions were given in total; two or three per ethnic group. In two educational meetings for the African-Surinamese participants the main themes of the community education could not be addressed completely (see further details in the results section). Therefore, we did one extra meeting for this ethnic group. Table 3 shows the characteristics of the meetings.

### 2.2. Analysis

Interviews with educators and participants, and the group discussions, were analyzed thematically [36,37]. XV and JS coded two interviews separately, after which codes were discussed and adjusted. Codes were grouped into overarching themes previously determined and based on the goals of the educational meetings and interview guides, such as ‘knowledge’, ‘wishes and needs for the last phase of life’, ‘insight in the importance of talking about the last phase of life’, ‘execution’ and ‘experience’. ‘Wanting to still enjoy life’ was for example a code related to ‘insight into the importance of talking about the last phase of life’. Outcomes from the different evaluation methods were compared among each other per session to check for accuracy of the findings, and between the sessions to find similarities and differences. Descriptive analysis was used for the questionnaires. Furthermore, the differences we found between the groups in the observations and group discussions, were analyzed by checking demographics that could logically relate to these differences, such as level of education.

### 2.3. Ethics

The medical ethical committee of the Amsterdam University Medical Centers/University of Amsterdam declared that this study did not require their approval, according to the Dutch Medical Research Involving Human Subjects Act [38]. We followed the ethical principles for medical research involving human subjects of the Declaration of Helsinki adopted by the World Medical Association [39]. We informed participants of the aims of the study and their legal rights. Educators gave written informed consent prior to the interview. Participants gave oral informed consent, because obtaining written informed consent led to mistrust in the first meeting. Individually interviewed participants gave written informed consent. We used codes to guarantee participants’ anonymity.

## 3. Results

### 3.1. Characteristics and Reach of the Educational Meetings

In total 136 participants joined the educational sessions, of which thirteen persons joined or left halfway. 91 (66,9%) participants filled in the questionnaire completely, 23 (16,9%) participants filled it in partly. Table 3 shows characteristics of the meetings. 97% of the participants was born in Suriname, Turkey or Morocco. Educational level differed highly between groups and within some groups (see Table 2). Most of the participants (58,4%) came to gain more knowledge about the last phase of life, but 35,2% of the participants also had others reasons, such as coming together with the group or just interest in what it would be about. 33% of the participants took care of someone in the last phase of life or had done this in the past.

### 3.2. Execution and Experience of the Community Educational Meeting

#### 3.2.1. Experience Measured by the Questionnaire

86% of the participants scored the provided information as good or excellent. 86% of the participants experienced talking about what would be important for yourself and doing this in a group as good or excellent. 92% of the participants scored the interactive elements (videos, questions, propositions) as fair/good, good, or excellent. On average, 88% of the participants experienced the meetings as good or excellent. No differences were found regarding the experience of the community education, as measured by the questionnaire, between the groups, or based on educational level, sex or age.

#### 3.2.2. Type of Information in Combination with the Interactive Method Was New

For many participants the information provided, about the palliative phase as a whole and the care aspects of it, in combination with an interactive manner was new (quote #1–3). Most groups were familiar with other educational sessions about specific illnesses (cancer, diabetes) or health care, by the same educator, but those were mainly informative and could be presented in a more light-hearted manner (quote #4). The levels of knowledge and speed of learning differed between and within groups. Within some groups repetition of information helped participants better grasp the content (meeting #1, #2, #4). Other groups, or certain people within a group, asked for additional information about medical choices (meeting #3, #6, #8).

Interviewer: *Do you think the education met the expectations and needs of the people in the room*? Respondent: *Yes, many things were new. In the beginning, the lady that sat here, said she wanted to know more about organizations and where to go to for help. And also family matters, what they already felt would be coming.* (#1, educator).

Interviewer: *Did the meeting bring you something new?* Respondent: *Yes, definitely. The videos were impressive and recognizable. The video in which the children are worried and have to make choices for the parents has stayed with me. That is what was in my mind and it is also important. It was new to watch this and to reflect on it.* (#2, participant).

*I discussed it elaborately with my family and partner. … They were in shock, because this is not an everyday subject. It is a hard subject and we don’t speak much about the last phase of life.* (#3, participant).

*Because, normally, education is about breast cancer or menopause. You can laugh about it and make jokes. This time it was really different. It is not really a subject they were waiting for to discuss.* (#4, educator).

#### 3.2.3. Overcoming Resistance Towards the Theme

Observations and interviews with the educators showed that, because of this interactive and confronting nature the educators had to overcome some resistance of participants, or groups, to talk about this subject (meeting #1, #2, #4, #5). However, these also seem to be the groups with lower median education level (see Table 2). Some participants, especially in the beginning, mentioned the subject being too hard, their children knowing more about it anyway and their life being in the hands of God/Allah as reasons for not wanting to talk about or plan for the last phase of life (quote #5). By using examples and videos, the importance of the subject became more clear and the participants were inclined to start talking about the subject themselves (quote #6). For the educators themselves the theme and/or the way they had to present it, was also new and they needed time to reflect and prepare for it. One of them mentioned her own inner constraint of setting herself to the subject at hand. In other groups participants started talking themselves about experiences regarding the last phase of life without needing to probe them. In most groups, eventually, personal experiences and emotions were unlocked and shared with the group. In one gender-mixed group however (meeting #5), it was mainly men who took the floor.

Interviewer: *For example, the opinions (of participants) were ”we will see how it will go”, “it is in Allah’s hands, in Allah’s hands” and “there is nothing we can do about it”. Indeed, they throw the towel in the ring again, “Let’s be done with this, it will come as it will.”* Educator: *Then, I intervene, “Yes we are Muslim, but.” Interviewer: They are not mutually exclusive.* Educator: *“Keep faith, but Allah doesn’t tell you that you are not allowed to think.”* (#5, educator).

*Then fear, it is discussed. But, if everyone would have said “I am not anxious”, then the rest would also have said ‘not anxious’. … So I said, “I don’t think about this subject, but I am anxious.” So then you (the participants) will think: “See! Shall I give my opinion?”* (#6, educator).

#### 3.2.4. Facilitating and Steering the Discussion

Observations and interviews with the educators showed all educators facilitated open discussion and a safe space for participants to share their opinions. Most educators also made sure several standpoints on the subjects were provided or shared by participants. They shared unpopular or vulnerable opinions and experiences of themselves, or asked critical questions about consequences of certain standpoints of participants. For example, they explained consequences of not wanting to know anything about diagnosis of prognosis, difficulty of accepting a diagnosis, differing opinions within the family, the added value of home care facilities and informal care support, or the consequences of the informal caregiver having to do everything him-/herself. (quote #7–#10).

*Knowing about how long you still have to live, in the beginning they were like “I don’t want to know that”. But they [the doctors] will tell you about it. That is reality. I think it is important to make them aware about reality*. (#7, educator).

*I support home care, but I try to not just bring my own opinion, or let it [my opinion] dominate. … But I explained about the possibility of incontinence and the need to be washed by someone else. “Who would you like to take up that task?” “Is it more convenient that a family member or home care worker does it?” Benefits and disadvantages. I told them that there are many possibilities they can make use of, not just the family or informal caregivers*. (#8, educator).

*That man* [a participant] *mentioned “culture” … The importance of cultural support in the last phase of life, and that things* [should] *happen according to culture. … And then I could respond, “That is ok, but what about the opinion of the person concerned?”* (#9, educator).

Interviewer: *You gave many examples from practice, that is your strength. You also were the first to mention about informal caregivers. … “You are at home with seven, but it will all come down to one person.” … It opens the discussion*. (#10, educator).

Educators differed to what extent they took a clear standpoint themselves and how well they illustrated these vulnerable or unpopular viewpoints and steered the discussion towards the main topics of the community education. Educators with extensive experience with community education about related subjects were better able to steer the conversation and come up with appealing examples or experiences themselves. One educator did not come up with these examples at all (meeting #7 and #8) and also didn’t elaborately address the main subjects. Another educator was not really able to do it the first time (meeting #5), and we—educator, observer and researcher XV – discussed afterwards the possible ways to do this the next time, which was successful. Fortunately, certain relevant opinions and themes were raised by participants themselves, but weren’t deepened by these educators Educators (educator #1, #2, #4) mentioned the large load of preparation and having to do many things at once: leading a group discussion, working with technology and educating about a new subject (quote #11).

Educator: *What I think is less [convenient], is using devices. … Then I have to look, how do I have to do it? I have to get used to that kind of thing. Interviewer: It is many things at once.* Educator: *And also with the group, giving the presentation and at the same time*. (#11, educator).

#### 3.2.5. Tools for Interaction

All educators used the videos with re-enacted family situations regarding weather to know or not to know about a diagnosis and prognosis, and the importance of family care. The situations were very recognizable for participants. The videos made them aware of the different opinions of involved family members and the consequences of those different opinions (quote #12). The videos evoked group discussions, and we also observed that they incited participants, who didn’t say much before, to speak up about their opinions or experiences. Voting on propositions was done in five meetings to foster interaction. One educator said it made the subject a bit more light-hearted (quote #13). Participants were actively involved while discussing the questions we set up to discuss in pairs, when actually discussed in pairs and not only plenary. The latter was done in some meetings.

Educator: *Then everything I had said came back in the videos. They saw a really sick person. They saw the experience. The mother who stood up for her daughter, who had to work continuously. The conflict between the brother and sister.* Researcher: *I had the feeling it made them pause for thought …* Educator: *The videos encompass so much information that will come back in real life*. (#12, educator).

*With those cards, green* [cards] *and red* [cards]. *It is quite something, but it makes it a bit more light-hearted and more fun*. (#13, educator).

### 3.3. The Added Value of the Community Educational Sessions

#### 3.3.1. Increase in Knowledge

One of the aims was to increase participants knowledge about palliative care and the palliative phase. We found that the educational sessions gave the participants an additional perspective on the last phase of life. Many participants were used to talk about the last phase of life from a religious perspective in their mosques, churches or community and did only mention religious aspects of the last phase of life or aspects of the very last days or moments after dying, both in the individual interviews, as well as in the group discussions. Others, sometimes within the same group, seemed to know, or spoke, about more practical or social aspects of the last phase and brought up possible loneliness and choices such as resuscitation and euthanasia. Participants of all the groups mentioned, in the group evaluation afterwards, that they learned about the professional- and care aspects of palliative care (quote #14), their legal rights, and that palliative care starts earlier than realized before.

Interviewer: *Was it important for you?* Respondent: *Yes, there are many things we don’t know about.* Interviewer: *You mean regarding palliative care?* Respondent: *Yes, you are sick and you don’t know if any help will come.* Interviewer: *Home care, for example.* Respondent: *Exactly, possibility of home care, we don’t know much about it*. (#14, participant).

Quite some interviewed participants showed more knowledge about the last phase of life in the interviews two-three weeks after the educational sessions. Some explained better what palliative care is and its aim to improve quality of life (compare quote #15 (before) and #16 (afterwards)), or mentioned specific palliative care services which they couldn’t mention before. Others were better able to put their knowledge about the existence of certain end-of-life choices such as euthanasia, which they already knew about beforehand, in the context of how such choices would take place in real-life situations.

Interviewer: *In that time [when her loved one was ill], did you hear anything about palliative care?* Respondent: *No, In that time you heard nothing. He received radiotherapy, we cared well for him.* Interviewer: *You didn’t hear about palliative care?* Respondent: *No never, we heard from Ayisha* (fictitious name of educator). *Something about helping people in the last phase of life*. (#15, participant).

Interviewer: *Could you tell me when and how the phase of palliative care starts?* Respondent: *Yes, it begins with taking good care of someone who cannot recover anymore and good care if someone does not have family. …* Interviewer: *When exactly do we think about palliative care?* Respondent: *Yes, in the last phase right? When someone doesn’t have long to live anymore. Or when a physician tells you that you will only have a couple of months to live.* Interviewer: *What do you mean, how ill? When does it start?* Respondent: *Yes, in the last phase of someone who is chronically ill. Or someone is anxious and cannot recover anymore.* (#16, participant).

#### 3.3.2. More Comprehensive Articulation of Wishes and Needs

Another expected outcome was that participants would be better able to describe their own wishes for the last phase of life; several participants did describe them more elaborately afterwards. One of these wishes concerned if and to what extent family members should take care of them and how their family could be supported better. Many participants already mentioned beforehand that their children would not be able to do all the caregiving, but only as a general statement. In two meetings, the idea of their own nursing home (or department), in which attention to their cultural and religious preferences is paid and volunteers from their own community would work, came up and was described more elaborately by participants in the group discussion afterwards (quote #17). Also, some individually interviewed participants showed a more elaborate view on caregiving; one described how she could take care of her mother with the help of other care services, another mentioned the actual positive shared experiences of children who still take care of elderly who live in a nursing home (compare quote #18 (before) and #19 (afterwards)). Other participants became aware of the fact that they didn’t want to know everything about their situation and that this could be a wish to act upon and that they have the right to not know and could tell their doctor about this wish and their legal rights.

Respondent 1: *We would like to have a commune, with capacity for people.* Respondent 2: *For people who are (work) in care can respond to. Imagine, I will become demented and I want to speak Hindu, but you cannot understand me, so we have to pay attention to it. There needs to be attention for that*. Respondent 3: *And also doing a lot of charity work. Because, in such a commune and there are not enough, we are with a lot. We have to do it with way more people*. Respondent 4: *But home care* Respondent 5: *A commune with care* Respondent 6: *And then everyone can help a bit*. Respondent 7: *Yes, with cleaning, vegetables, flower, decorating*. Respondent 8: *You will come and see that others are helping.* (#17, group discussion afterwards).

Respondent: *Trying to keep living like this and not being a burden to others. I want to keep living a good life, but not become a burden to others*. Interviewer: You don’t want others. Respondent: *Have to do for me what I cannot do myself. So no resuscitation, or I would want to go immediately. Or I *[have to]* live, but care for myself*. (#18, participant).

Respondent: *About the collaboration between elders and younger people, was positive. I can learn something form that, that younger people still treat their elders rightly.* Interviewer: *And also how they do it?* Respondent: *I thought it was beautiful to see how people nowadays.. in the past you were obliged to take care of your parents. …* Interviewer: *So if it would be needed [for him]*? Respondent: *If it is needed, yes then. I have two daughters, so I hope they will*. (#19, participant).

#### 3.3.3. Awareness and Opening Up About Their Own Situations

Another aim was to increase insight into the importance of talking about this subject. While the themes and interactive methods were a little confronting, many participants valued this as something positive at the end of the meeting. Many participants emphasized in the group evaluation afterwards and the individual interviews the importance of talking about the last phase of life in an educational session like this one. The provided information combined with the opportunity to hear experiences of others in the group (quote #20), made them pause for thought and led to insight about what to expect and what to think about themselves. Some participants (#1, #7) explicitly mentioned they talked about their own situation in these sessions, while they normally don’t do or dare to do this. One participant (#7) mentioned not to be aware before that the situation with their relative might be a palliative phase already (quote #21). For some (#2), the meeting brought up memories of people that passed away and existing worries about the last phase of life; talking about this subject was therefore also hard.

Interviewer: *Was it valuable for you?* … Respondent: *Yes, you also learn from others and they learn something from you. It is always about experiences. There was also someone who was just in the middle of it …* Interviewer: *A realistic view on the situation?* Respondent: *Yes. Interviewer: And that is the added value, why you would rather do it in a group*? Respondent: *Yes, preferably*. (#20, participant).

*Then I saw (the video) and thought to myself, when the girl told about how hard it was to bring her mother there (to the nursing home), “I just say it.” We didn’t come (to the educational meeting) for this. … It is a phase of life you forget about. My husband is there (the nursing home) already for so long, 11 years, but he will also. When someone is sick, then it is mourning and that is also a phase of life, a goodbye. I am thinking, “What did I arrange for him?” I didn’t even think about that*. (#21, group discussion afterwards).

#### 3.3.4. Differing Opinions About the Moment of Talking About the Last Phase: Right Now, or In the Future

Most of the interviewed participants did not change their opinion on the importance to talk about the last phase of life with close others far before the palliative phase. This didn’t meant they didn’t discuss anything. Some already thought it was important to discuss wishes regarding the last phase of life, most others discussed their wishes for the last moments or after dying (quote #22). Some participants did clearly state the importance of talking about wishes and making a plan in time (#1, #2, #3) because they did not want to bother the patient with it in a difficult stage (quote #23), but did not have plans to do so in the near future. Others (#3, #5, #6, #7, #9) would want, or trusted, to be able to discuss their wishes with their loved ones when the time is there (quote #24). They mentioned the last phase of life as something far away, and they wanted to enjoy life as long as it is still possible and therefore not bother themselves or close one’s with difficult questions at this moment.

Respondent: *I told my brother and sisters* [about my own funeral], *“Let my wife decide on how she wants to do it” I don’t want others to meddle. I welcome all help, but I want them to do it like she wants it to.* Interviewer: *This is about the funeral?* Respondent: *Yes. Because now, there are so many different traditions.* (#22, participant).

*No, with someone who is in their last phase, you should not come up with these subjects. When someone doesn’t start about it themselves, others should not bring it up.* (#23, participant).

*The moment you encounter it [the last phase of life], then explain everything clearly. What would be the possibilities and effects of certain decisions. All that kind of things. It can be said, it should not be concealed.* (#24, participant).

## 4. Discussion

Participants and educators experienced the provided information about the last phase of life as new and, in combination with encouragement to talk about it themselves, somewhat confronting. All educators were eventually able to overcome the existing initial resistance to talk about this subject, or the slow pace of exchange of experiences. Educators did this by using lively illustrated examples of their own lives or practices, showing videos with enacted real-life situations and questions that critically accessed participants’ opinions to show the importance of the subject and to ensure that diverse standpoints were addressed. They facilitated an open discussion to share experiences and evoked group discussion. Some educators had to grow into this role, or didn’t bring the main subjects across elaborately.

We evaluated the added value of the educational meetings we co-created with the target groups, on the three preferred outcomes—increased knowledge about palliative care, increased comprehensiveness of descriptions of wishes and needs regarding dignity in the last phase of life and increased insight into the importance of talking about your wishes—of which the first two were effectively increased among participants and the third one partly. Additionally, for many participants the experienced added value was to learn about this subject, the care aspects of the last phase, their legal rights, that palliative care starts earlier than realized, and that they talked about subjects that were important to them. Participants were mainly used to talk about the last phase of life from a religious perspective, or just about the phase after dying. The provided information combined with the opportunity to hear experiences of others, led to insight about what to expect and provided them with the opportunity to talk about things they normally wouldn’t talk about.

Earlier research mainly focused on increasing knowledge regarding advanced care planning [22,23,24], or gaining trust of the minority community by assuring care professionals would not talk about death or pain with them [40], because discussing death was considered a taboo for these patients [17]. Our study showed it is possible to have a group conversation about the last phase of life with migrants despite an initial resistance towards the theme [41], and it showed a prior existing lack of awareness that the palliative phase encompasses a larger phase rather than the last weeks or moments. Educators with the same ethnic background and experience and familiarity with themes within the community, were able to bridge an initial resistance. Our study showed that talking about palliative care is possible, if done sensitively and with examples and themes that are recognizable and important for participants.

The origin of the initial resistance might be a cultural one and may also be related to the role of religion in participants’ lives or to religious coping, as some participants used religious reasons, such as trusting God or Allah, to not plan for the future or talk about the future. Resistance may also be related to lack of knowledge, participants’ own confidence to actually talk properly about this subject, or their level of self-efficacy [42]. The groups in which it took longer to get the conversation going, were also the groups with a lower education level median. Volandes and colleagues comparably found low health literacy, which is related to low educational level [43], to be associated with a preference for aggressive advanced care decisions regarding advanced dementia. A video with a patient with advanced dementia, in contrast to verbal explanations about dementia, abated all differences regarding aggressive end-of-life preferences associated with race and health literacy [44]. This implies there may be a similar communication barrier in end-of-life preferences and the initial resistance to talk about the last phase of life in our study: the use of vivid examples that embody situations and videos with re-enacted real-life situations changed initial perspectives.

The literature proposes community engagement as a way for capacity building in palliative care and better outcomes of interventions. Our study is—to our knowledge—one of the first evidence-based studies of such an approach in palliative care [17,45]. We complied to important elements of community engagement [46] (see box 1) and were therefore able to reach many participants, and execute community education in which participants felt at ease; at the same time educators developed skills to discuss this in their communities. By focusing on themes relevant for migrant communities our community education was responsive to concerns of migrant patients and their families, increased their knowledge about palliative care and descriptions of their wishes and needs in the palliative phase. In order to warrant good quality community engagement, we recommend that researchers, partner organizations and educator(s) discuss together which candidates could lead group discussions with a certain goal and transcend their own experience to address, and dare to address, diverse positions and situations; and provide support for growing into this role.

Generalizability of the findings may be limited, because a large amount of the participants were women. This was partly because we worked together with female educators and because of a preference for single-gender groups among Turkish and Moroccan participants, but also in the other groups we were not able to recruit as much men as women. Additionally, women are more often the primary informal caregiver in their family which may incited them to participate in this education. We may need more male recruiters, or need to encourage men in another way, for them to be interested in participating in community education about the last phase of life. Several studies found gender differences regarding preferences for (emotional) support in the last phase of life or during depression [47,48]. Future research could investigate whether such gender differences indeed exist across various ethnic backgrounds or minority populations and how these can be facilitated in community education.

Following the Stages of Change Model [49], we can see community education as a first step towards improved access to palliative care services, capacity building and a dignified last phase of life among migrants. Participants gained more knowledge and moved from pre-contemplation to contemplation about how to act upon their needs and wishes, and some towards actual preparation. Participants gave more comprehensive descriptions about their wishes and needs for a dignified last phase of life, and also new thoughts came up within the groups which could be developed further in follow-up meetings. A second step would involve to train more educators who are able to train other key community stakeholders, so that participants actually start discussing what they have learned with their close ones and care professionals and may even start acting on ideas generated together within such meetings. This actual change of behavior may be fostered by having follow-up meetings, or by splitting up the educational meeting in several shorter sessions to let participants digest the material and remind them about the subject.

## 5. Conclusions

Community education about palliative care for migrants is effective when developed in collaboration with, and carried out by, educators with the same ethnic backgrounds who know their communities well. Our education, which included topics especially important for dignity of migrant patients, effectively increased knowledge about palliative care and increased awareness about the palliative phase starting much earlier than realized and about what would be important for a dignified last phase of life for themselves. All three components seem to be needed for migrants to be able to act upon wishes and needs and improve utilization and experience of palliative care, and hence improve access to palliative care services, whether this includes complete utilization of care services or assistance of family who gives care themselves. Our study shows it is possible to overcome an initial resistance towards talking about the last phase of life with vivid examples and videos with re-enacted situations, and familiar educators. It also shows an existing need among migrants to talk about their experiences under guidance of an educator with knowledge of the topic and a lack of knowledge that the last phase of life encompasses a larger phase than just the last days of life. The provided information combined with the opportunity to hear experiences of others in the group were experienced as valuable by the participants. Community engagement and our community education can be a first step towards improved access to palliative care services and a dignified last phase of life among migrants.

## Figures and Tables

**Table 1:** Methods for evaluation.

Method	Who Is Studied	What It Measures or Explores	Related Research Question	When	Number of Participants	Duration (min)	Place
Semi-structured Interview	Educator	Experience, execution	1, 2	Directly after the educational meeting	8, 1 per group	30–45	Place of educational meeting
Semi-structured Interview	Participant	Level of knowledge, wishes regarding the last phase of life, insight in the importance of talking about the last phase of life	2	Before the educational meeting	8, 1 per group	20–30	Place of educational meeting
Semi-structured Interview	Interviewed participant (see 2)	Experience, level of knowledge, wishes regarding the last phase of life, insight in the importance of talking about the last phase of life	2	2–3 weeks after the educational meeting	8, 1 per group	30–50	At home, community center or by phone
Questionnaire	Participant	Experience of provided information and interactive elements, demographics, reach	1	Partly before and partly directly after the educational meeting	All participants	n.a.	Place of educational meeting
Observation	Participant and educator	Responses and interaction of participants, execution by educator	1	During the educational meeting	All educational sessions	120	Place of educational meeting
Group acquaintance	Participant	Familiarity with subject: knowledge about palliative care and experience with talking about it.	2	Before the educational meeting	8, 1 per group	10–15	Place of educational meeting
Group evaluation	Participant	Experience, experienced added value	2	Directly after the educational meeting	8, 1 per group	10–15	Place of educational meeting

**Table 2:** Characteristics of educators.

Nr.	Age	Sex	Country of Birth	Educational Level	Religion
#1	41	F	Turkey	University	Islam
#2	36	F	Morocco	Intermediate professional education	Islam
#3	47	F	Morocco	Intermediate professional education	Islam
#4	69	F	Suriname	Higher professional education	Hinduism
#5	-	F	Suriname	University	Evangelical brotherhood church
#6	57	F	Suriname	University	Catholicism

**Table 3:** Characteristics of the educational meetings.

	Ethnic Background	Amount of Participants	Age Range	Female/Male Ratio	Used Language for Execution	Education Level Median (IQR of 6p-Likert Scale)	Place
#1	Turkey	9	54–60 (+ one of age 16)	9/0	Turkish	1 = Primary school (3)	Home care organization
#2	Turkey	8	51–76	8/0	Turkish	1 = Primary school (0)	Home care organization
#3	Morocco	14	36–58	14/0	Dutch	3 = Intermediate professional education (1)	Mosque
#4	Morocco	14	40–73	14/0	Berber/Arabic	1 = Primary school (1)	Nursing home
#5	Suriname-South Asian	21	60–90	16/4	Dutch	1,5 = Primary school/secondary school (1)	Hindustani-elderly commune
#6	Suriname-South Asian	27	61–83	21/6	Dutch	2,5 = Secondary school/Intermediate professional education (2)	Community center
#7	Suriname-African	21	59–77	11/1	Dutch	4 = Higher professional education (0)	Community center
#8	Suriname-African	6	66–80	2/4	Dutch	4 = Higher professional education (2)	Surinamese-elderly commune
#9	Suriname- African	21	60–88	19/2	Dutch	3 = Intermediate professional education (2)	Community center

## Supplementary Materials

The following are available online at https://www.mdpi.com/1660-4601/17/21/7797/s1. Some of the videos with re-enacted real life situations in the last phase of life are available online at https://ingesprek.pharos.nl/.

## Appendix A. Questionnaire

### Questionnaire Prior to the Meeting

1. What is your reason for participating in this meeting? (multiple answers possible) □ The topic is relevant for me. □ I want to know more about the last phase of life. □ I am thinking about the last phase of life myself. □ Others told me it would be good to join. □ Other reason:	
2. How good is your health?	
3. How much do you trust your doctor will give you good care?	
4. Do you get help with dressing or other daily activities by a family member or nurse?	□ Yes, daily □ Yes, weekly □ Yes, monthly □ Rarely □ Never
5. Do you think it is useful to talk about the last phase of life?	

Questionnaire afterwards

1. What do you think about the videos we used?	
2. What do you think about the given information about palliative care?	
3. What do you think about the propositions we talked about? (if used)	
4. What do you think about the questions we asked to talk about the last phase of life?	
5. How did you experience to talk about what would be important for you during the last phase of life?	
6. How did you experience it to do this in a group setting?	
7. How did you experience it to talk about family care?	
8. What did you think about the place (at which this meeting was held)?	
9. Do you live far from this place?	Yes
	No
10. What did you think about the educator?	
11. What did you think about the group composition?	
12. Do you take care of someone in the last phase of life, or did you do this in the past?	Yes
	No

We would also like to ask you some questions regarding your personal information. Your personal information will be stored in a safe place which only the researchers have access to. The personal information will be anonymized. Your name will not be asked for.

1. How old are you? …………. years.
2. I am a
  □ man
  □ woman

3. What is your religion?
  ………..…………..…………..…………..…………..…………..…………..………………………..

4. In which country were you born?
  …………..…………..…………..…………..…………..…………..…………..………………………..

5. In which country was your father born?
  …………..…………..…………..…………..…………..…………..…………..………………………..

6. In which country was your mother born?
  …………..…………..…………..…………..…………..…………..…………..………………………..

7. Which education did you take?
  □ None
  □ Primary school
  □ Secondary school
  □ Intermediate professional education
  □ Higher professional education
  □ University

8. What kind of job do you do?
  ………………………………………………………………………………………………………………

## Appendix B. Interview Guides

### B.1. Individual Interview with Participant Prior to the Meeting

Personal information

Age:
  ……………………………………………………………………………………………………………………
Sex:
  ……...……………………………………………………………………………………………………………
Religion:
  ……………………………………………………………………………………………………………………
Country of birth:
  ……………………………………………………………………………………………………………………
Country of birth father:
  ….………………………………………………………………………………………………………………
Country of birth mother:
  ……………………………………………………………………………………………………………………
Educational level:
  ……………………………………………………………………………………………………………………
Occupation:
  ……………………………………………………………………………………………………………………

Introduction: You will participate in a meeting about the last phase of life. In this meeting you will receive information about healthcare and we will talk together about the meaning of the last phase of life.

Could you tell me why you will be attending this meeting?

1. Did you talk with others about the last phase of life before?
  Probe:
  - What did you talk about?
  - Do you know someone who is in his/her last phase of life?

2. Do you think it is useful to talk about the last phase of life? Why is it, or isn’t it?
3. What do you know about palliative care? What is palliative care according to you?
  Probe about:
  a. Do you know the goal of palliative care?
  For example: improve quality of life, relief suffering, minimize symptoms
  b. When does palliative care (or the last phase of life) start?
  For example: Once you get diagnosed with a life-threatening disease
  c. What type of care do you expect palliative care to encompass?
  For example: medical, social, psychological and spiritual care

4. Do you know about which choices you could encounter in the last phase of life?

For example: wanting to know about diagnose or prognosis, who you would like to take care of you, who should take decisions for you in case you cannot do it yourself, morphine or palliative sedation, resuscitation, continuation of nutrition, euthanasia, religious aspects or rules, how to say goodbye, cremation or funeral, legacy.

5. Do you know which type of care you could use in the last phase of life?

For example: home care, nursing home, informal care support, personal budget (PGB), hospice (last weeks), 24-h care at home (last weeks), professional translation service.

6. Do you know about what would be important for you in the last phase of life? Probe about what would be important.
7. Do you know who you would like to take care of you? Probe about who and why.
8. Did you talk about the last phase of life before with a care professional?

### B.2. Individual Interview with Participant 2–3 Weeks Afterwards

1. How did you experience the educational meeting?
2. Did it do you good to talk about this subject with other participants?

a. What was valuable?

3. Did it bring you new perspectives? (effectivity)

a. Did your opinion change because of what was discussed during the meeting?

4. Could you tell me what palliative care is?
  Ask additional questions:
  a. Do you know the goal of palliative care?
  For example: improve quality of life, relief suffering, minimize symptoms
  b. When does palliative care (or the last phase of life) start?
  For example: Once you get diagnosed with a life-threatening disease
  c. What type of care do you expect palliative care to encompass?
  For example: medical, social, psychological and spiritual care

5. Do you know about which choices you could encounter in the last phase of life? For example: wanting to know about diagnose or prognosis, who you would like to take care of you (family or others), who should take decisions for you in case you cannot do it yourself, morphine or palliative sedation, resuscitation, continuation of nutrition, euthanasia, religious aspects or rules, how to say goodbye, cremation or funeral, legacy.

6. Do you know which type of care you could use in the last phase of life? For example: home care, nursing home, informal care support, personal budget (PGB), hospice (last weeks), 24-h care at home (last weeks), professional translation service.

7. Do you think it is useful to talk about the last phase of life? Why is it, or isn’t it?
8. Do you know what would be important for you in the last phase of life?
  a. What would you need?
  b. Would you want family members to take care of you? Why, or why not?

9. Did you talk about the last phase of life with someone else after the educational meeting? Why, or why not?
  If so.
  How did you experience it? What did you discuss?
  With whom did you talk about it?
  If not, what would be helpful to do it?

10. Did you discuss your wishes with a physician or other professional caregiver? Why, or why not? What did you discuss?

### B.3. Group Discussion with Participants Prior to the Meeting

*Researcher and educator do this together.*

We would like to know what your experience is with the last phase of life.

1. What do you have in mind when you think about the last phase of life? Which subjects are you concerned with?
2. What do you expect to hear in this meeting today?
3. Do you ever talk with others about the last phase of life? What do you discuss?

### B.4. Group Discussion with Participants Afterwards

Researcher and educator do this together

We would you like to know how you experienced the educational meeting.

1. What did the meeting bring you? What was valuable?
2. What did you experience as pleasant? Was there also something you experienced as difficult?
3. What would you like to know more about?

### B.5. Interview with Educator

*Researcher and research assistant do this together*

Introduction: I would like to shortly evaluate the educational meeting and how you experienced it. I would also like to hear you observations.

Topics

1. How did it go?
2. What did you experience as pleasant?
3. What did you experience as difficult?

Probe about

- What the participants expected
- What needs participants had
  i. Information needs
  ii. Subjects they wanted to discuss
- Participation level of participants
  i. Feeling comfortable, daring to share their opinions, effect of the interactive elements we used
  ii. Understanding of provided information among participants

4. Why did you execute the meeting the way you did it? Discuss notable observations.
5. Which themes provoked discussion among participants?
6. Do you have additional things you would like to mention about the educational meeting and your experience?

Personal information

Age:         …………………………………………….
Sex:         …………………………………………….
Religion:      ……………………………………………….
Country of birth:     ………………………………………….
Country of birth mother: ……………………………………….
Country of birth father: ………………………………………….
Occupation:      ………………………………………….
Educational level:   …………………………………………….

## Appendix C. Observation Checklist

### Researcher and Research-Assistant do This Together

Meeting

Date:                …………………….
City:                …………………….
Place (mosque, nursing home etc.):    …………………….
Target group (ethnic background):    …………………….
Amount of participants:        …………………….
Language:               …………………….

Topics

Check fidelity: Is the education executed as discussed and described?

- Correct use of PP-presentation
- Are the most important subject addressed completely? (see schedule)
- Is the order of the script followed?
- Stimulation of conversation among participants

○ How does the educator do this?
○ Do participants get enough time to think about it?
○ Do educators address several perspectives?

Check participation, appropriateness and responsiveness

- Are participants engaged in the conversation? Why/when are they and when aren’t they?
- Do the participants seem to be comfortable?
- Do they participate during the interactive parts?
- Do the participants seem to understand the information? Do they ask probing questions?
- Do the subjects of the educational meeting speak to the participants and what they would like to discuss about the last phase of life themselves?

Additional themes?

- Are there additional themes the participants start to discuss, or would like to discuss?
