# Supplementary material for: Community Education for a Dignified Last Phase of Life for Migrants: A Community Engagement, Mixed Methods Study among Moroccan, Surinamese and Turkish Migrants
Source: Int J Environ Res Public Health. 2020 Oct 24;17(21):7797. doi: 10.3390/ijerph17217797 (PMC7662901; doi:10.3390/ijerph17217797)
Supplement: Supplementary file 1 [file ijerph-17-07797-s001.pdf]

Codetree interviews individual participants		
**Theme**	**Codes**	**Subcodes**
Motivation to participate		
	Obtaining knowledge	
	It is an important subject	
	Coming together with the group	
Experience of the meeting		
	Appropriateness	Confronting subject
		Good to have more awareness
		Being able to discuss all possibilities
	Responsiveness	Knowledge / information about palliaitve care (services) was needed
		Relevant subjects discussed (family care)
	Doing this in a group (was beneficial)	
	Bring in own knowledge	
Importance of the educational meeting		
	Lack of information by care professionals	
	Arranging a dignified/good last phase of life	
	Knowledge need	
Own wishes and needs		
	Before / No wishes because of unfamiliarity	
	Before / Respect for the dead	
	Before / Practicing religion	
	Before / Being cared for as long as possible	
	Before / Not wanting to become a burden	

	Before / Giving value to life
	Before / No pain or extension of suffering
	Before / Not experiencing the last phase
	Before / Wishes regarding after dying: cremation or funeral
	Before / Utilize external care
	Before / Deciding myself about the end of life
	Before / Weather wants to know about diagnosis/prognosis
	After / Practicin religion
	After / Not wanting to become a burden
	After / Weather wants to know about diagnosis / prognosis
	After / Wishes regarding after dying: cremation or funeral
	After / Affection during the last days
	After / Wishes regarding medication and medical choices
	After / Loving care
	After / Receiving information
	After / Religious- and culture sensitive care
	After/ Utilize external care
Knowledge about palliative care	Before / No knowledge becuase of unfamiliarity
	Before / Start of the palliative phase
	Before / Medication and medical choices
	Before / Palliative care services
	Before / Person-centered care
	Before / General discription of palliative care or it's definition
	After / Start of the palliative phase

After / Medication and medical choices
After / Palliation and loving care
After / Palliative care services
After / Person-centered care

The importance of talking about the last phase of life

Talking with whom

Close family or friends
With a care professional

Motivations or reasons to do/or not to do

Before / Expecting to be led through by a physician

Before / (grand)Children don't like to talk about the topic

Before / Having the time to talk about this subject

Before / The last phase of life is still far away

Before / Not wanting to burden close others with this subject

Before / Everyone has to experience it one day

Before / My mind is not on it
Before / Discussing only who decides about this subject

After / Too charged subject
After / Not appropriate to talk about this when the phase is there

After / Trusting choices or knowledge of (close) others

After / My mind is not on it

		After / Trusting network for knowing where to go when the time is there
		After / Still wanting to enjoy life
		After / The last phase of life is still far away
		After / Being prepared
		After / (Trusting to being able to) talk openly about when the time is there
**Codetree interviews educators**		
Educator’s skills and struggles	Difficulties with probing or directing the discussion	
	New type of subject for the educators.	Notes: not just sending information, getting the group to talk about this subject, overcoming own inner hurdle to talk and think about this subject, breaking a culture or resistance towards it.
Aspects of the education itself	Multitude of preparations and actions	
Degree of participation	Differences between participation of participants	
	Many participants participated	
Eliciting opinions and experiences of participants	Asking critical questions and probing on opinions.	Notes: discussing pro’s, con’s consequences and why’s
	Educator gives examples (that embody situations)	Notes: from their medical or personal experience

	Responsiveness towards additional knowledge need of participants	
	Giving space for several opinions	
	Educator first let participants share general experiences about palliative care	
	Showing own vulnerability of the educator	
Giving information about palliative care (services)	Telling people how they could benefit from palliative care services	
	Taking clear standpoints about what the doctor will do	
	Repetition of information needed	
Noticed experience/responses of participants	Participants wanted to talk about it.	
	Participants shared their experiences.	
	Emotional subject for people who lost someone	
	Confronting subject	Notes: some distanced themselves from the subject (using the belief in Allah to not talk about this subject) or said to trust their children.
Interactive methods	Videos had recognizable situation and conflicts of real-life situations	
	Videos made the group calm and pause for thought	
	Videos evoked group discussion	
	Voting yes/no made it more light-hearted	
